# Supplementary material for: Pseudogene Transcripts in Head and Neck Cancer: Literature Review and In Silico Analysis
Source: Genes (Basel). 2021 Aug 17;12(8):1254. doi: 10.3390/genes12081254 (PMC8391979; doi:10.3390/genes12081254)
Supplement: Supplementary file 1 [file genes-12-01254-s001.zip › genes-1299320-supplementary.pdf]

## Supplementary Information

# Pseudogene Transcripts in Head and Neck Cancer: Literature Review and *In Silico* Analysis

**Juliana Carron [1], Rafael Della Coletta [2] and Gustavo Jacob Lourenço [1,*]**

[1] Laboratory of Cancer Genetics, School of Medical Sciences, University of Campinas, Campinas, SP, 13083-888, Brazil; julianacarron@outlook.com.br
[2] Department of Agronomy and Plant Genetics, University of Minnesota, Saint Paul, MN 55108, USA; della028@umn.edu
* Correspondence: guslour@unicamp.br, Tel.: +55-19-3521-9120

**Citation:** Carron, J.; Della Coletta, R.; Lourenço, G.J. Pseudogene Transcripts in Head and Neck Cancer: Literature Review and *In Silico* Analysis. *Genes* **2021**, *12*, 1254. https://doi.org/10.3390/genes12081254

Academic Editor: Deborah J. Good

Received: 29 June 2021
Accepted: 12 August 2021
Published: 17 August 2021

**Publisher's Note:** MDPI stays neutral with regard to jurisdictional claims in published maps and institutional affiliations.

**Copyright:** © 2021 by the authors. Submitted for possible open access publication under the terms and conditions of the Creative Commons Attribution (CC BY) license (http://creativecommons.org/licenses/by/4.0/).

**Table S1.** Clinicopathological aspects of 219 head and neck cancer patients selected at The Cancer Genome Atlas database.

| Characteristics | Number of Patients (Range or %) |
|---|---|
| **Median age (years)** | 60 (38-89) |
| Gender | |
| Male | 174 (79.5) |
| Female | 45 (20.5) |
| Ethnic origin | |
| White | 183 (83.6) |
| Non-white | 30 (13.7) |
| Not reported | 6 (2.7) |
| Tumor localization | |
| Oral cavity | 62 (28.3) |
| Oropharynx | 51 (23.3) |
| Hypopharynx | 8 (3.7) |
| Larynx | 98 (44.7) |
| Vital status | |
| Alive | 130 (59.4) |
| Dead | 89 (40.6) |

**Table S2.** Most deregulated pseudogenes in head and neck cancer and its subtypes identified at The Cancer Genome Atlas database, genetic variation type and identifier.

| Pseudogene Transcript | Genetic Variation Type (n) | Genetic Variation Identifier (ID) |
|---|---|---|
| *SPATA31D5P* | SNV (19) | chr9:g.81917110T>C; chr9:g.81916294C>T; chr9:g.81916788C>G; chr9:g.81915701G>A; chr9:g.81916112A>T; chr9:g.81917939C>T; chr9:g.81917426G>T; chr9:g.81918866C>T; chr9:g.81916813G>C; chr9:g.81917603C>T; chr9:g.81917898C>T; chr9:g.81916693T>C; chr9:g.81916906G>A; chr9:g.81915617C>T; chr9:g.81915633G>A; chr9:g.81917204T>A; chr9:g.81919502C>A; chr9:g.81917927C>A; chr9:g.81916688C>A |
| *HERC2P3* | SNV (19) | chr15:g.20383438A>T; chr15:g.20439375G>T; chr15:g.20452449G>T; chr15:g.20452462G>A; chr15:g.20452555G>A; chr15:g.20438651T>C; chr15:g.20383431C>T; chr15:g.20453853C>A; chr15:g.20440493G>A; chr15:g.20438683G>A; chr15:g.20452505C>A; chr15:g.20439040C>A; chr15:g.20438824T>G; chr15:g.20453634C>T; chr15:g.20383366C>T; chr15:g.20444372C>T; chr15:g.20444256G>T; chr15:g.20444227C>A; chr15:g.20444371C>T |
| *SPATA31C2* | SNV (15) | chr9:g.88132450G>T; chr9:g.88131173G>T; chr9:g.88134788G>A; chr9:g.88131242A>C; chr9:g.88131995T>G; chr9:g.88131928G>T; chr9:g.88132087G>T; chr9:g.88131854G>C; chr9:g.88131333C>G; chr9:g.88132406G>A; chr9:g.88132101C>T; chr9:g.88132278C>A; chr9:g.88131939A>C; chr9:g.88132182G>T; chr9:g.88129720C>T |
| *MAGEB6P1* | SNV (15) | chrX:g.26160667C>G; chrX:g.26161441G>A; chrX:g.26160625C>A; chrX:g.26161623C>T; chrX:g.26161408C>A; chrX:g.26161374C>G; chrX:g.26160629G>C; chrX:g.26161687G>T; chrX:g.26161039G>T; chrX:g.26161010G>T; chrX:g.26161091C>G; chrX:g.26161454C>G; chrX:g.26160651G>A; chrX:g.26161152T>A; chrX:g.26161707G>C |
| *SLC25A51P1* | SNV (13) | chr6:g.65788530C>T; chr6:g.65788587A>G; chr6:g.65789220G>T; chr6:g.65788451C>G; chr6:g.65788893C>A; chr6:g.65788561C>T; chr6:g.65788602C>T; chr6:g.65788894T>G; chr6:g.65788704T>A; chr6:g.65789088G>T; chr6:g.65788563C>A; chr6:g.65789247C>A; chr6:g.65788770A>T |
| *BAGE2* | SNV (12) | chr21:g.10413565C>A; chr21:g.10473718C>A; chr21:g.10473325G>T; chr21:g.10473449T>C; chr21:g.10473596C>A; chr21:g.10413636G>A; chr21:g.10473632G>A; chr21:g.10413730G>T; chr21:g.10473316C>A; chr21:g.10473241C>A; chr21:g.10413587G>T; chr21:g.10454138G>A |
| *DNM1P47* | SNV (8) | chr15:g.101759775G>T; chr15:g.101762730C>T; chr15:g.101752519C>G; chr15:g.101759755C>G; chr15:g.101764268G>A; chr15:g.101753184G>T; chr15:g.101759784C>A; chr15:g.101752987G>C |
| *SPATA31C1* | SNV (13) and del (1) | chr9:g.87922410C>T; chr9:g.87920997C>G; chr9:g.87919151T>A; chr9:g.87920595C>A; chr9:g.87921094C>T; chr9:g.87922031G>T; chr9:g.87920433G>A; chr9:g.87922796G>T; chr9:g.87921535G>T; chr9:g.87920604C>A; chr9:g.87920350G>A; chr9:g.87922158C>A; chr9:g.87921534G>T; chr9:g.87921488delG |
| *ZNF733P* | SNV (10) | chr7:g.63292321G>T; chr7:g.63292374T>A; chr7:g.63292245G>A; chr7:g.63292191C>A; chr7:g.63292378C>T; chr7:g.63291761C>A; chr7:g.63292237G>A; chr7:g.63292132G>T; chr7:g.63292812G>T; chr7:g.63291599C>A |
| *OR2W5* | SNV (10) and del (1) | chr1:g.247491764C>T; chr1:g.247491713G>T; chr1:g.247491194C>A; chr1:g.247491778C>T; chr1:g.247491919A>G; chr1:g.247491820C>A; chr1:g.247491316T>G; chr1:g.247492077G>A; chr1:g.247491717C>A; chr1:g.247491708T>C; chr1:g.247491293delCA |
| *NBPF25P* | SNV (7) | chr1:g.145578886T>C; chr1:g.145578049G>A; chr1:g.145586293C>G; chr1:g.145587982G>A; chr1:g.145587966C>A; chr1:g.145580723C>T; chr1:g.145574113G>C |
| *NXF4* | SNV (7) | chrX:g.102550109G>C; chrX:g.102562668C>T; chrX:g.102563371G>T; chrX:g.102564000G>A; chrX:g.102550112G>A; chrX:g.102567021C>T; chrX:g.102568467G>T |
| *BNIP3P1* | SNV (7) | chr14:g.28264892C>G; chr14:g.28265090G>T; chr14:g.28264729C>T; chr14:g.28264677G>C; chr14:g.28264503C>T; chr14:g.28264837C>A; chr14:g.28264618A>G |
| *PKD1L2* | SNV (2) | chr16:g.81174810G>T; chr16:g.81175654G>C |
| *ZNF658B* | SNV (7) | chr9:g.39445654G>A; chr9:g.39446976C>A; chr9:g.39445373G>A; chr9:g.39447267G>A; chr9:g.39446313G>A; chr9:g.39445270C>A; chr9:g.39445269A>C |
| *POTEA* | SNV (6) | chr8:g.43292781G>T; chr8:g.43292519C>T; chr8:g.43318555C>G; chr8:g.43318595C>T; chr8:g.43318569C>T; chr8:g.43318547G>C |

| | | |
|---|---|---|
| *MROH5* | SNV (8) | chr8:g.141507213G>A; chr8:g.141490163G>A; chr8:g.141435999C>G; chr8:g.141476053C>G; chr8:g.141496335C>T; chr8:g.141472881A>T; chr8:g.141440315C>T; chr8:g.141435204G>A |
| *MSL3P1* | SNV (2) | chr2:g.233867311G>C; chr2:g.233867189C>G |
| *HLA-H* | SNV (2) | chr6:g.29888008G>C; chr6:g.29889597G>C |
| *TUBB8P7* | SNV (5) | chr16:g.90095366G>T; chr16:g.90095367C>T; chr16:g.90094640G>A; chr16:g.90095655C>T; chr16:g.90095622G>A |
| *SLC7A5P2* | SNV (2) | chr16:g.21519961G>A; chr16:g.21519895G>C |
| *DPY19L2P1* | SNV (5) | chr7:g.35090359T>C; chr7:g.35104687G>A; chr7:g.35104704C>T; chr7:g.35090332A>T; chr7:g.35090346T>C |
| *TSSC2* | SNV (3) | chr11:g.3407576G>A; chr11:g.3401855C>T; chr11:g.3401768C>G |
| *DPY19L2P3* | SNV (5) | chr7:g.29694607C>A; chr7:g.29687396G>A; chr7:g.29697009T>G; chr7:g.29733553G>A; chr7:g.29742234T>A |
| *GBA3* | SNV (8) | chr4:g.22736146G>T; chr4:g.22747855A>G; chr4:g.22747913G>A; chr4:g.22727581A>T; chr4:g.22747778C>A; chr4:g.22693025C>A; chr4:g.22748026T>A; chr4:g.22818740C>G |
| *PLEKHM1P* | SNV (4) | chr17:g.64800344T>C; chr17:g.64800567G>A; chr17:g.64797321G>A; chr17:g.64792411C>T |
| *MST1P2* | SNV (4) | chr1:g.16643086C>T; chr1:g.16649682G>C; chr1:g.16646409C>A; chr1:g.16649657G>C |
| *ADAM21P1* | SNV (1) and ins (1) | chr14:g.70246544C>G; chr14:g.70247179_70247180insT |
| *OR12D2* | SNV (6) | chr6:g.29397288C>A; chr6:g.29397420G>T; chr6:g.29396714C>G; chr6:g.29397313C>G; chr6:g.29396774C>T; chr6:g.29397334C>T; |
| *PNLIPRP2* | SNV (8) | chr10:g.116626969G>C; chr10:g.116626952T>A; chr10:g.116626910G>A; chr10:g.116626924G>T; chr10:g.116634889A>T; chr10:g.116636825T>C; chr10:g.116623984A>T; chr10:g.116636905A>G |
| *HSP90AB2P* | SNV (5) | chr4:g.13337246G>A; chr4:g.13336782A>G; chr4:g.13336798C>A; chr4:g.13338268G>A; chr4:g.13337610G>A |

number of genetic variations, SNV: single nucleotide variation, del: deletion, ins: insertion.
